# Conventional versus hypofractionated postmastectomy radiotherapy: a report on long-term outcomes and late toxicity

**DOI:** 10.1186/s13014-019-1378-x

**Published:** 2019-10-14

**Authors:** Imjai Chitapanarux, Pitchayaponne Klunklin, Attapol Pinitpatcharalert, Patumrat Sripan, Ekkasit Tharavichitkul, Wannapha Nobnop, Wimrak Onchan, Somvilai Chakrabandhu, Bongkot Jia-Mahasap, Juntima Euathrongchit, Yutthaphan Wannasopha, Tanop Srisuwan

**Affiliations:** 1 Division of Radiation Oncology, Faculty of Medicine, Chiang Mai University, 110 Intawarorose Road, Chiang Mai, 50200 Thailand; 2 Northern Thai Research Group of Radiation Oncology (NTRG-RO), Faculty of Medicine, Chiang Mai University, Chiang Mai, Thailand; 3 Chiang Mai Cancer Registry, Maharaj Nakorn Chiang Mai Hospital, Faculty of Medicine, Chiang Mai University, Chiang Mai, Thailand; 4 Division of Radiation Oncology, Faculty of Medicine, Thammasat University, Bangkok, Thailand; 5 Department of Radiology, Faculty of Medicine, Chiang Mai University, Chiang Mai, Thailand

**Keywords:** Long-term, Outcome, Toxicity, Conventional, Hypofractionated, Postmastectomy radiotherapy

## Abstract

**Objective:**

We evaluated the long-term outcomes and late toxicity of conventional fractionated (CF) and hypofractionated (HF) postmastectomy radiotherapy (PMRT) in terms of locoregional recurrence-free survival (LRRFS), disease-free survival (DFS), overall survival (OS), and late toxicity.

**Methods:**

A cohort of 1640 of breast cancer patients receiving PMRT between January 2004 and December 2014 were enrolled. Nine hundred eighty patients were treated with HF-PMRT: 2.65 Gy/fraction to a total of 42.4–53 Gy and 660 patients were treated with CF-PMRT: 2 Gy/fraction to a total of 50–60 Gy.

**Results:**

The median follow-up time was 71.8 months (range 41.5–115.9 months). No significant difference was found in the rates of 5-year LRRFS, DFS, and OS of HF-PMRT vs CF-PMRT; 96% vs. 94% (*p* = 0.373), 70% vs. 72% (*p* = 0.849), and 73% vs. 74% (*p* = 0.463), respectively. We identified a cohort of 937 eligible breast cancer patients who could receive late toxicities assessment. With a median follow-up time of this patient cohort of 106.3 months (range 76–134 months), there was a significant higher incidence of grade 2 or more late skin (4% vs 1%) and subcutaneous (7% vs 2%) toxicity in patients treated with HF-PMRT vs CF-PMRT. Patients who received additional radiation boost were significantly higher in the HF-PMRT group. Grade 2 or more late RTOG/EORTC lung toxicity was significant lesser in HF-PMRT vs CF-PMRT (9% vs 16%). Grade 1 brachial plexopathy was also significant lesser in HF-PMRT vs CF-PMRT (2% vs 8%). Heart toxicity and lymphedema were similar in both groups.

**Conclusions:**

HF**-**PMRT is feasible to deliver with comparable long-term efficacy to CF-PMRT. HF-PMRT had higher grade 2 or more skin and subcutaneous toxicity but less lung and brachial plexus toxicity.

## Introduction

Radiotherapy is one of the essential components of breast cancer treatment. After a mastectomy, radiotherapy as an adjuvant treatment has shown benefits in both locoregional control (LRC) and overall survival (OS) in many large randomized clinical trials and meta-analysis [1–4]. Generally, the standard radiation dose for postmastectomy radiotherapy (PMRT) is 50 Gy with 2-Gy daily fractions over 5 weeks.

Since the 2000s, hypofractionated radiotherapy (HFRT) schemes with a fraction size of more than 2 Gy have been used in breast cancer treatment. Although this radiotherapy schedule has the benefit of reducing the overall treatment time, late toxicity is of concern. Based on radiobiological data, the healthy breast and underlying structure tissues are sensitive to the fraction size, volume irradiated, and total dose delivered [5]. Hence, even slightly higher radiation doses per fraction can result in severe late toxicity. Despite concerns about late tissue toxicity, many large studies have proved that hypofractionation radiotherapy after breast-conserving surgery is harmless. The updated results of a landmark trial from Canada has shown that using a regimen of 42.5 Gy in 16 fractions after breast-conserving surgery to treat early stage breast cancer provides locoregional control, OS, and cosmetic outcomes similar to the standard radiotherapy regimen at 10-years of follow-up [6]. Another two large randomized controlled trials from the UK have also confirmed that using HFRT regimens (41.6 Gy in 13 fractions, 39 Gy in 13 fractions, and 40 Gy in 15 fractions) are safe [7, 8]; the authors concluded that a lower total dose in a smaller number of fractions could offer rates of tumor control and normal tissue damage similar to the conventional fractionation schedule of 50 Gy in 25 fractions. Additionally, their 10-year follow-up report revealed that in the START-A trial, moderate or marked breast induration, telangiectasia, and breast edema were significantly lower in the 39 Gy group, and in the START-B trial, breast shrinkage, telangiectasia, and breast edema were significantly lower in the 40 Gy group than in the 50 Gy group. Thus, they encourage the continued use of 40 Gy in 15 fractions as the standard of care for invasive early breast cancer treatment [9].

Hypofractionated postmastectomy radiotherapy (HF-PMRT) has been implemented in many institutes. However, the data on its efficacy and side effects are still sparse. Since our institute serves a large number of breast cancer patients and the radiotherapy waiting time is quite long, we have been using HF- PMRT since 2004. The aim of this retrospective study is to report on the long-term outcomes of HF-PMRT and CF-PMRT in terms of locoregional recurrence-free survival (LRRFS), disease-free survival (DFS), OS, and late toxicity.

## Material and method

### Patients

We reviewed 2457 medical records of breast cancer patients who received radiotherapy at our center between 2004 and 2014. We assessed the diagnosis, stage, side of breast cancer, status of the disease, information about the treatment, and late toxicity. Breast cancer patients who had undergone mastectomy and required adjuvant PMRT to the chest wall with or without axilla and supraclavicular lymph nodes were included.

The treatment techniques for all cases were applied by using either 2D or intensity-modulated radiotherapy (IMRT) treatment planning. In the case of 2D treatment planning between 2004 and 2011, the 2D plans were performed using two methods: computerized tomography (CT) images and manual contouring of the breast. The chest wall was treated by using two tangential parallel opposed fields. The borders of the two tangential beams were determined clinically and the chest wall was manually contoured or CT-scanned via a single axial slice. The dose distributions were calculated only on the central single slice. Wedges were used to improve the tissue dose homogeneity and all patients were treated with a cobalt-60 beam. A 0.5-cm bolus provided an adequate build-up of the dose on the skin surface during the first half of the treatment course. From 2012 to 2014, all patients were treated with a 6-MV photon beam after 2D or IMRT treatment planning. In the case of 2D planning, two tangential beams were determined on the CT scan in the single axial slice with a wedge for beam modification. A 1-cm bolus was used to build up the dose on the skin surface during the first half of the treatment course. The bolus was used in all patients who treated with 2D technique irradiation which balanced patient’s number for CF-PMRT (100%) and HF-PMRT (99%). Bolus used for part of the treatment course for full skin dose, needing removal for the latter half of treatment and requiring two treatment plans to be generated, one for the bolus and one for the non-bolus fractions. Regarding IMRT treatment planning, all dose distributions ware calculated on the full CT slices. After installing a helical tomotherapy (HT) unit in March 2012 at our center, we started treating some breast cancer patients with this machine. The HT parameter definitions were 2.5 and 5 cm for the field width, 0.287 and 0.215 for the pitch, and 2.5–3.5 for the modulation factor.

The hypofractionation schedule used at our center comprised 2.65 Gy per fraction and one fraction per day, the same as that used in the Canadian Study [6] to 16 fractions or 18-20 fractions in positive margins or T4d disease according to American Joint Committee on Cancer staging (AJCC), while the conventional schedule was 2 Gy per fraction, 5 fractions per week to 25 fractions or 28-30 fractions in positive margins or T4d disease. The additional boost dose was applied to the whole thoracic wall in both CF and HF PMRT groups. Patients with a locoregionally recurrent tumor or metastatic disease at diagnosis were excluded. Other exclusion criteria were if patients had received treatment at other radiotherapy centers, had received fractionation other than the conventional schedule or 2.65 Gy per fraction and 5 fractions per week, had undergone breast-conserving surgery, had received prior radiotherapy to the breast or chest wall, had not completed radiotherapy as planned, and/or had an incomplete medical record. Eligible patients received a mailed invitation to follow-up together with a questionnaire about late radiation toxicity. We analyzed late toxicity in two patient cohorts: the first comprised any patient who had an archived medical record mentioning late toxicity (which had occurred after 6 months or more post-PMRT), while the second comprised patients who could come to follow-up at the time of the analysis and were free from locoregional recurrence and lung metastasis to prevent obfuscation of the symptoms between those two situations.

The Radiation Therapy Oncology Group (RTOG)/ European Organization for Research and Treatment of Cancer (EORTC) Late Radiation Morbidity Scoring Schema was the most commonly used at our center [10]. Although we found that late skin and subcutaneous tissue toxicity had been graded according to the schema in the medical records, the late toxicity of lung, cardiac, brachial plexus, and arm edema had not. Since we retrospectively gathered information from medical record which did not mention about grading severity of toxicity, we only had evidence of the presence or absence (YES/NO) of the symptoms of these late toxicity occurrences in the normal tissues in the first cohort patients. Therefore, for those patients in the second patient cohort who were available for follow-up received an actual late toxicity assessment; we used the RTOG/EORTC Late Radiation Morbidity Scoring Schema for grading the late toxicity of skin, subcutaneous tissue, lung, and heart. For arm lymphedema, we used lymphedema staging by the International Society of Lymphology (ISL) [11]: stage 0 is the subclinical stage and swelling is not seen despite underlying changes in the lymphatic system, stage 1 is the initial stage of swelling which can be transient and simple elevation can alleviate the edema, stage 2 is constant swelling and pitting without resolution using elevation, and stage 3 in which tissue has become hard and fibrotic with associated skin changes. Brachial plexopathy was assessed by modified LENT SOMA scales [12]: grade 1- mild sensory deficit, no pain, and no treatment required; grade 2 - moderate sensory deficit, tolerable pain, and mild arm weakness; grade 3 - continuous paresthesia with incomplete paresis for which pain medication is required; and grade 4 - complete paresis, excruciating pain, and muscle atrophy for which regular pain medication is required.

LRRFS was calculated from the date of surgery until the date of recurrence in the ipsilateral chest wall, supra/infraclavicular regions, axilla, and internal mammary region, which were diagnosed by a physical examination or imaging. Pathological examination for recurrent disease was performed in some cases. DFS was calculated from the date of surgery until the date of the first relapse or the last follow-up. OS was calculated as the period of time from the date of surgery to the date of death from any cause or the date of the last follow-up. Survival times were censored at the date of last contact for patients who did not follow-up. The status of the patients and the date of death were obtained by the mortality data from the National Registration Department.

### Statistical analysis

The data was locked on March 31st 2019. Descriptive analyses were summarized as medians with range or interquartile range (IQR) for non-normally distributed continuous characteristics. Frequencies and proportions were reported for categorical characteristics. Patient and treatment characteristics and late toxicity were compared for the two treatment schema group using Wilcoxon Rank Sum test for non-normally distributed continuous characteristics and the Fisher’s exact test for categorical characteristics. Time to events was compared between two treatment schema group by Kaplan-Meier survival procedure and Log Rank test. The *p* value reports were two-tailed with an alpha level of 0.05 for statistical significance. All analyses were conducted using Stata version 11 (StataCorp LP, College Station, TX, USA). This study was approved by the Research Ethics Committee of Faculty of Medicine, Chiang Mai University.

## Results

**Fig. 1:**
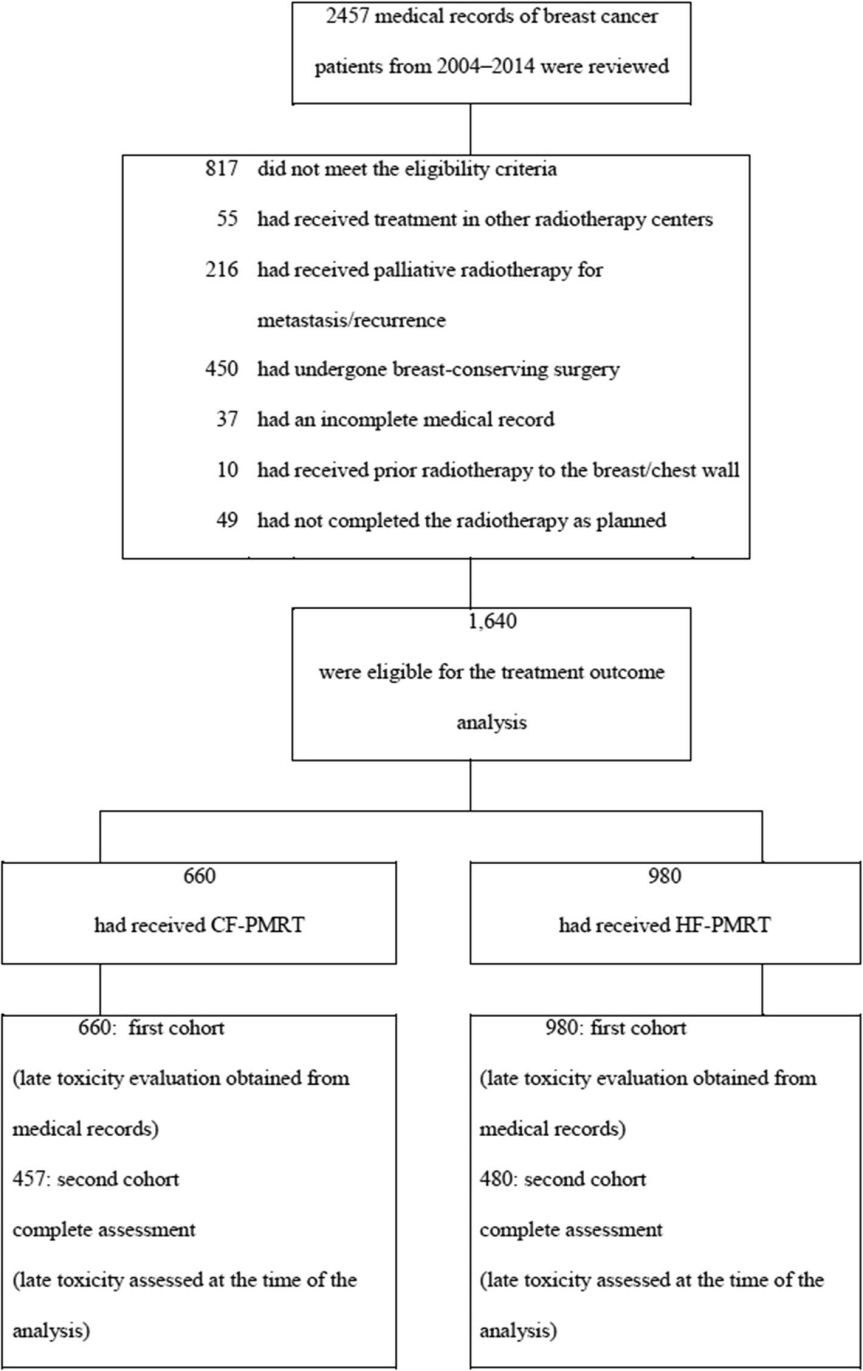
A flow chart of the study population

One thousand six hundred forty patients met the eligibility criteria for the evaluation of treatment outcomes. Of these, 660 patients (40.2%) were received CF-PMRT and 980 patients (59.8%) were received HF-PMRT. Patient and treatment characteristics are reported in Table 1. When comparing both schedules, the HF-PMRT group was significantly younger, at an earlier stage (stages I and II), less likely to have received chemotherapy, less likely to have received regional nodal irradiation, and more likely to have received additional boost dose of radiotherapy than the CF-PMRT group (*p* < 0.001). Invitation letters to follow-up together with the questionnaires on late toxicity were sent to all of the patients. Only 937 (57.1%) were eligible for follow-up and were enrolled in the second patient cohort for the assessment of late toxicity at the time of analysis: 457 patients in the CF-PMRT group and 480 patients in the HF-PMRT group. Toxicity assessment was completely evaluated up to 98% of second patient cohort for both treatment groups. Late toxicity grading was evaluated as the worst grade of symptoms persisting for more than 6 months after the end of PMRT. A flow chart for selecting the study population is shown in Fig. 1.

**Table 1:** Patient and treatment characteristics

Variable	CF-PMRT n (%) *N* = 660	HF-PMRT n (%) *N* = 980	*p*-value
Age (Median; IQR) (year)	57 (49–64)	51 (45–58)	< 0.001^*^
Tumor side			0.011
Left side	324 (49)	492 (50)	
Right side	323 (49)	484 (49)	
Both sides	13 (2)	4 (1)	
AJCC stage			< 0.001
I	32 (5)	54 (6)	
II	329 (50)	559 (57)	
III	250 (38)	323 (33)	
IV	14 (2)	44 (4)	
Unknown	35 (5)	0 (0)	
Chemotherapy			0.027
No	23 (3)	58 (6)	
Yes	637 (97)	922 (94)	
Hormonal therapy			0.257
No	248 (38)	397 (41)	
Yes	412 (62)	583 (59)	
Radiotherapy technique			0.001
2D technique with bolus	660 (100)	967 (99)	
Tomotherapy	–	13 (1)	
Radiotherapy technique			< 0.001
CW only	20 (3)	90 (9)	
CW plus RNI			
- CW + SPC	453 (69)	595 (61)	
- CW + SPC+ Axillary	187 (28)	295 (30)	
Radiotherapy boost			< 0.001
No	554 (84)	731 (75)	
Yes	106 (16)	249 (25)	

*CF-PMRT* Conventional fractionated post mastectomy radiotherapy, *HF-PMRT* Hypofractionated post mastectomy radiotherapy, *N* number, *IQR* Inter Quartile Range, *AJCC* American Joint Committee on Cancer, *CW* chest wall, *SPC* supraclavicular, *RNI* regional nodal irradiation

*p*-value for Fisher’s exact except for ^*^Wilcoxon rank-sum (Mann-Whitney)

**Fig. 2:**
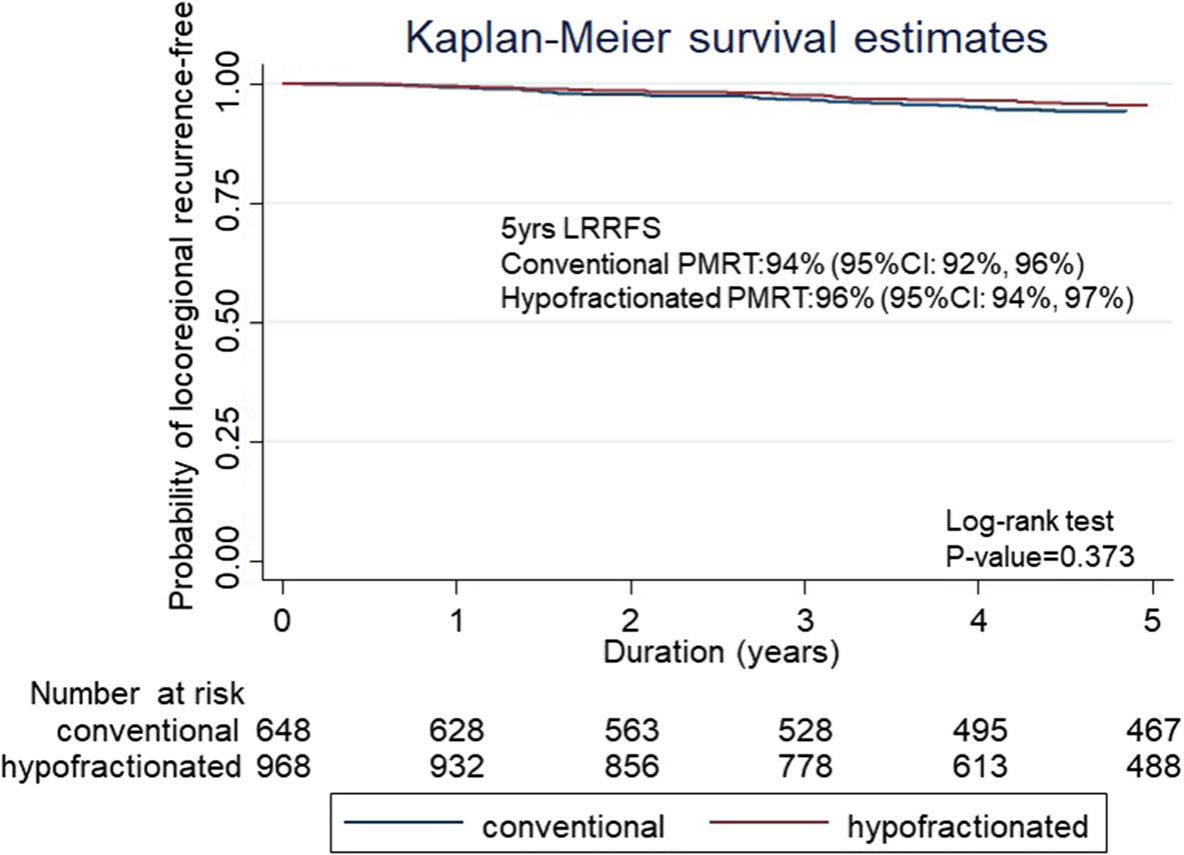
Kaplan-Meier curves of locoregional relapse free survival (LRRFS) for conventional fractionated and hypofractionated postmastectomy radiotherapy (PMRT)

**Fig. 3:**
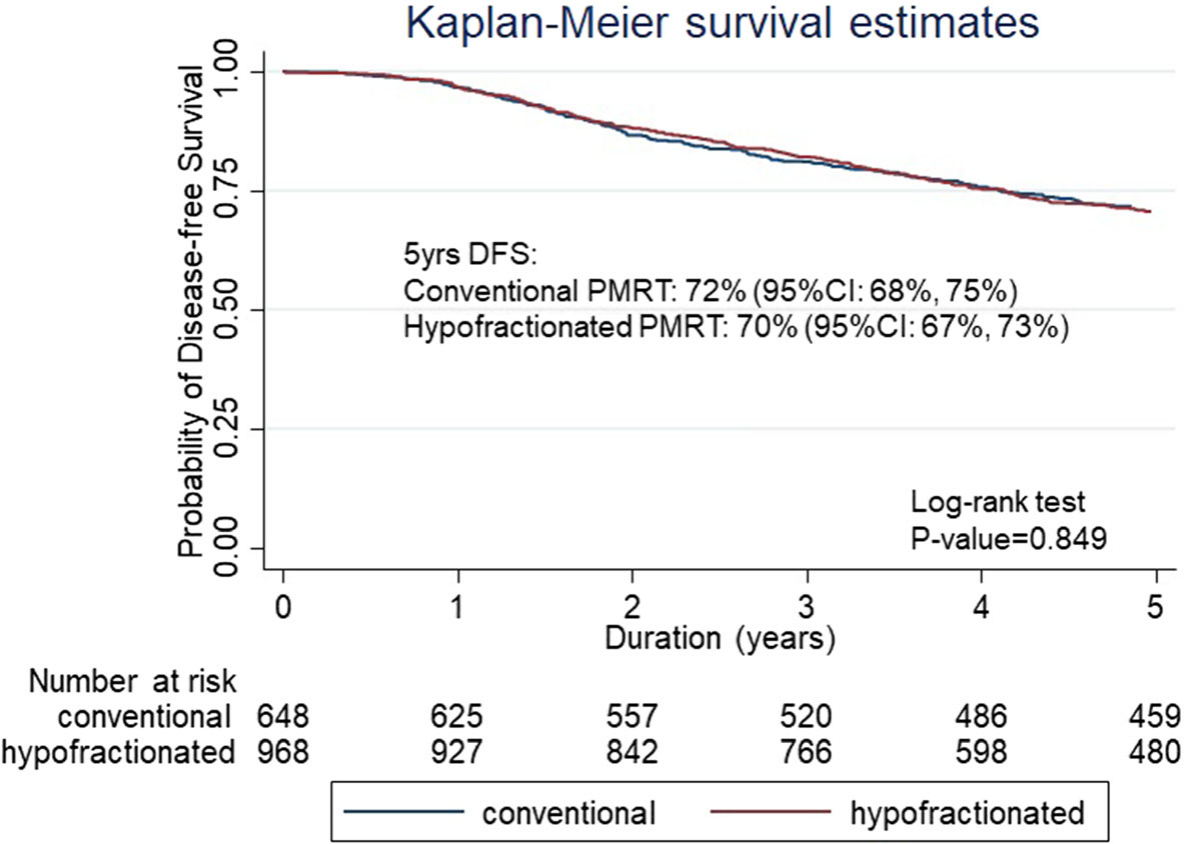
Kaplan-Meier curves of disease-free survival (DFS) for conventional fractionated and hypofractionated postmastectomy radiotherapy (PMRT)

**Fig. 4:**
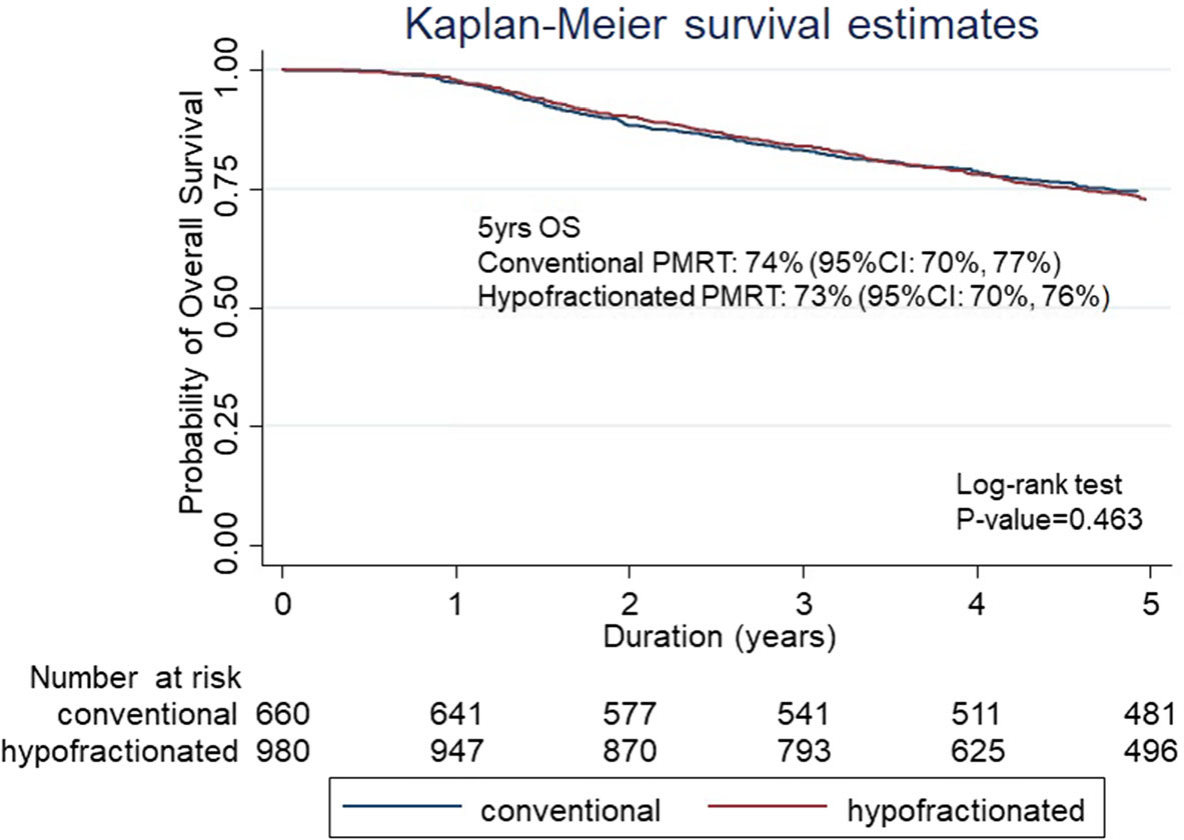
Kaplan-Meier curves of overall survival (OS) for conventional fractionated and hypofractionated postmastectomy radiotherapy (PMRT)

After a median follow-up of 71.8 months (range 41.5–115.9 months), the 5-year LRRFS rates for CF-PMRT versus HF-PMRT were 94% (95% confidence intervals (CI): 92–96) and 96% (95% CI: 94–97) with a *p*-value of 0.373 (Fig. 2). The 5-year DFS rates for CF-PMRT versus HF-PMRT were 72% (95% CI: 68–75) and 70% (95% CI: 67–73) with a *p*-value of 0.849 (Fig. 3). The 5-year OS rates for CF-PMRT versus HF-PMRT were 74% (95% CI: 70–77) and 73% (95% CI: 70–76) with a *p*-value of 0.463 (Fig. 4).

The late toxicity evidence for all 1640 patients obtained from their medical records (the first cohort) is summarized in Table 2. We found that HF-PMRT group was statistically significantly higher for the late toxicity of skin, subcutaneous tissue, and lung than in CF-PMRT group. The incidence of late cardiac and lymphedema toxicity was not different between the two treatment schedules. Next, we identified toxicity in the second cohort of 937 of eligible breast cancer patients who received an assessment of late toxicity with a median follow-up time of 106.3 months (range; 76-134 months). The late toxicities of both RT schedule groups in this patient cohort are given in Table 3. The incidence of severe late toxicity (grade 2 or higher) was very low in both schedules. However, we found a statistical significance of late RTOG grade ≥ 2 skin (4% versus 1%) and subcutaneous tissue toxicity (7% versus 2%) in the HF-PMRT and CF-PMRT groups, respectively. For late RTOG lung toxicity that was assessed by the clinical symptom, the incidence of grade 2 (persistent symptoms requiring symptomatic treatment) was very low at 1% or less in both groups. While EORTC lung toxicity which was assessed by imaging (patchy or increased density imaging changes), the incidence of grade 2 or more was higher in the CF-PMRT group. Consequently, the combining RTOG/EORTC severe grade of late lung toxicity was also found higher in CF-PMRT. Grade 1 brachial plexopathy was also found significant higher in the CF-PMRT. Lymphedema ISL stage 2 was observed in 4 patients in CF-PMRT (1%) and 4 patients in HF-PMRT (1%), with no statistically significant difference between the schedules.

**Table 2:** Late toxicity in the first cohort from patient medical records

Toxicity	CF-PMRT n (%) *N* = 660	HF-PMRT n (%) *N* = 980	*p*-value
Skin^a^			< 0.001
N	641	942	
Grade 0	276 (43)	482 (51)	
Grade 1	355 (55)	427 (45)	
Grade 2	10 (2)	23 (3)	
Grade 3	0 (0)	10 (1)	
Subcutaneous tissue^a^			< 0.001
N	641	942	
Grade 0	358 (56)	622 (66)	
Grade 1	267 (42)	225 (24)	
Grade 2	16 (2)	95 (10)	
Lung^b^			< 0.001
N	641	955	
No	539 (84)	473 (50)	
Yes	102 (16)	482 (50)	
Heart^b^			0.272
N	641	942	
No	630 (98)	932 (99)	
Yes	11 (2)	10 (1)	
Lymphedema^b^			0.072
N	640	942	
No	618 (97)	924 (98)	
Yes	22 (3)	18 (2)	

*CF-PMRT* Conventional fractionated post mastectomy radiotherapy, *HF-PMRT* Hypofractionated post mastectomy radiotherapy, *N* number

^a^RTOG/EORTC Late Radiation Morbidity Scoring Schema [11]

^b^no record of grading, information of any symptoms that related to the toxicities only

**Table 3:** Late toxicity in the second cohort from the assessment at the time of analysis

Toxicity	CF-PMRT n (%) *N* = 457	HF-PMRT n (%) *N* = 480	*p*-value
Skin^a^			< 0.001
Grade 0	197 (43)	340 (71)	
Grade 1	255 (56)	122 (25)	
Grade 2	5 (1)	9 (2)	
Grade 3	0 (0)	9 (2)	
Subcutaneous tissue^a^			< 0.001
Grade 0	261 (57)	370 (77)	
Grade 1	185 (41)	75 (16)	
Grade 2	11 (2)	35 (7)	
Lung (Clinical)^a^			< 0.001
Grade 0	389 (85)	239 (50)	
Grade 1	62 (14)	240 (50)	
Grade 2	6 (1)	1 (< 1)	
Lung (Imaging)^a^			< 0.001
Grade 0	184 (40)	249 (52)	
Grade 1	199 (44)	187 (39)	
Grade 2	24 (5)	7 (1)	
Grade 3	50 (11)	37 (8)	
Heart^a^			0.840
Grade 0	453 (99)	477 (99)	
Grade 1	0 (0)	0 (0)	
Grade 2	1 (< 1)	0 (0)	
Grade 3	3 (< 1)	3 (< 1)	
Lymphedema^b^			0.905
Stage 0	442 (97)	467 (97)	
Stage 1	11 (2)	9 (2)	
Stage 2	4 (1)	4 (1)	
Brachial Plexopathy^c^			< 0.001
Grade 0	421 (92)	469 (98)	
Grade 1	36 (8)	11 (2)	

CF*-PMRT* Conventional fractionated post mastectomy radiotherapy, *HF-PMRT* Hypofractionated post mastectomy radiotherapy, *N* number

^a^RTOG/EORTC Late Radiation Morbidity Scoring Schema [11]

^b^ISL [12]

^c^Modified LENT SOMA scales [13]

## Discussion

There have been several studies to investigate HF-PMRT in locally advanced breast cancer [13–20], mostly trials on retrospective data and using 2D radiotherapy techniques, as summarized in Additional file 1: Table S1. A trial of 300 randomized patients in Pakistan with three different hypofractionation regimens consisted of 27 Gy in 5 fractions in arm A, 35 Gy in 10 fractions in arm B, and 40 Gy in 15 fractions in arm C [13]. With a median time to follow-up of 60 months, the locoregional recurrences rate (LRR) were 11, 12, and 10%, respectively; 26, 24, and 28% developed distant metastatic disease (DM) and the specific mortality rates were 17, 18, and 20%, respectively. All of the end-points were not statistically significantly different between the three treatment regimens. A study in Egypt compared three different hypofractionated radiotherapy schedules: 50 Gy in 25 fractions (group A; 41 patients), 45 Gy in 17 fractions (group B; 36 patients), and 40 Gy in 15 fractions (group C; 30 patients) [14]. At the 7 year follow-up, no statistically significant difference in local recurrence (3, 1, and 2 patients, respectively) and mortality (4, 2, and 3 patients respectively) was found between the groups. Researchers in Greece compared conventional radiotherapy (50 Gy in 25 fractions) and hypofractionated radiotherapy (48.3 Gy in 21 fractions and 42.56 Gy in 16 fractions) and demonstrated that no patients developed a locoregional relapse with any of the treatment regimens [15]. However, this study had a short-term follow-up interval with a median time of 36 months. A study conducted at the Moroccan National Institute of Oncology retrospectively reviewed hypofractionated radiotherapy treatment at a dose of 42 Gy in 15 fractions [16]; they reported a 5-year OS rate of 87%, a 5-year DFS rate of 84%, and a 5-year LRC rate of 94%. Meanwhile, a prospective study was carried out in New Jersey after the delivery of a radiation dose of 36.63 Gy in 11 fractions to 69 patients [17]; the authors revealed 3% ipsilateral chest wall recurrence, an 89% 3-year LRC rate, and a 90.3% 3-year distant metastasis-free survival rate.

Recently, the first randomized trial comparing 2D conventional radiotherapy with a dose of 50 Gy in 25 fractions and 2D hypofractionated radiotherapy with a dose of 43.5 Gy in 15 fractions was conducted by a Chinese institute [18]; 810 patients participated with a median follow-up interval of 58.5 months. The results were comparable in both regimens in terms of 5-year OS rate (86% vs 84%), DFS rate (70% vs 74%), locoregional relapse (8% vs 8%), and 5-year distant failure (27% vs 23%). Indian study randomized 100 patients to CF-PMRT with a dose of 50Gy in 25 fractions and HF-PMRT with 42.7Gy in 16 fractions using 3D conformal radiotherapy technique. With a median follow up of 20 months, the rate of OS, DFS, LRR and DM were not significant difference between two radiotherapy schedules [19]. Our previous study compared a CF-PMRT group treated with 50 Gy in 25 fractions and an HF-PMRT group with 42.4 Gy in 16 daily fractions or 47.7 Gy in 18 fractions delivered every other day [20]; the 5-year LRC rates were 87 and 86% and the 5-year DFS rates were 63 and 70% in the CF-PMRT and HF-PMRT groups, respectively. These results are similar to the others, the exception being the unexplainable 5-year OS rate which was significantly higher in the HF-PMRT group (63 and 73% in the CF-PMRT and HF-PMRT groups, respectively). The present study has a much longer (nearly double) follow-up time compared to our prior report (71.8 months versus 39 months), and this time we found that the outcomes were comparable with the other studies [1–6] with no statistically significant differences in 5-year LRRFS, DFS, and OS between the two treatment schedules.

As mentioned before, only late skin and subcutaneous tissue toxicity were graded according to the RTOG/EORTC Late Radiation Morbidity Scoring Schema in the medical records, but not late toxicity to the lung, heart, brachial plexus, and arm edema. Any symptoms documented in the medical records related to late toxicity of such organs, i.e. coughing, dyspnea, fatigue, arm weakness, arm pain, and/or arm edema enabled us to assign the presence or absence of late toxicity in the related normal tissue. However, these symptoms might be indistinguishable if the patient had a recurrence of the disease, either locoregionally or as a distant metastasis. Thus, we tried to overcome this effect by inviting all patients via mail for a late toxicity assessment at the time of analysis. For the actual late toxicity evaluation in this second cohort, we excluded patients who had had a recurrence (either locoregional or distant metastasis) since this would obscure the symptoms of late toxicity, i.e. lung metastasis versus radiation pneumonitis and locoregional recurrence versus lymphedema. Hence, we could assess the late toxicities in only 57% of the patients, of which 69% were from the CF-PMRT group and 49% from the HF-PMRT group, which might indicate the strength of our study.

In addition, we had the longest follow-up both for treatment outcomes and late toxicity compared to the other studies [13–20]. The incidences of late radiation complication in the aforementioned trials are summarized in Additional file 2: Table S2. Our study demonstrates that patients who received HF-PMRT had statistically significantly higher late skin and subcutaneous toxicity than those who received CF-PMRT, which was the case in both the first and second patient cohorts, with the latter having a longer follow-up at a median of 106.3 months. However, HF-PMRT had a significant higher number of radiation boost patients than CF-HFRT (*p* < 0.001); the late skin toxicity is also increase corresponding with a high dose delivery. The incidence rate of severe skin toxicity (grade 2 or higher) was comparable to other studies [15, 18–20], except for the Egyptian study [14]. The Egyptian study published grade 2 or higher skin fibrosis of 17% with the conventional radiation group, 33% in the 45 Gy in 17 fractions group, and 37% in the 40 Gy in 15 fractions group [14]. Conversely, the study from Greece had no patients who developed grade 2 or higher late skin toxicity in both the CF-PMRT and HF-PMRT groups [15]. The Chinese study also found grade 3 late skin complications in less than 1% of the HF-PMRT group and 0% in the CF-PMRT group [18]. The Indian study reported 4% of grade 2 or higher chronic dermatitis rate for both CF-PMRT and HF-CRT [19]. Neither of these studies recorded a statistically significant difference between the groups. Our previous study by Pinitpatcharalert et al. demonstrated a grade 2 or higher skin toxicity of 9% in the CF-PMRT group and 10% in the HF-PMRT group and concluded that late skin toxicity was comparable between two treatment arms [20]. However, our previous study excluded histological margin-positive patients that need additional dose of radiation to avoid unequal biological effective dose (BED) between treatment arms whereas our recent study included the patients who received additional boost dose. Concerning about bolus application, radiotherapy treatments of post-mastectomy chest wall are complex, requiring treatment close to the skin, necessitating bolus use. Commonly used 5- and 10-mm-thick boluses develop full skin dose [21]. There are wide variations in the use of application of bolus in radiotherapy treatment. A worldwide survey carried out in 2004 by the Sunnybrook Health Sciences Centre, Canada [22], reviewed the use of bolus in PMRT for the US were significantly to always use a bolus (82%) than the Europeans (31%) for specific indications, as were the Australasian (65%). The Alternated day and thickness used 1.0 cm were the most application for PMRT [21].

With regard to cardiac toxicity, cardiovascular events were very low. In the Chinese randomized study, grade 1–3 ischemic heart disease was noted in only 1 and 2% in conventional and hypofractionated groups, respectively [18], which is not statistically significant. Pinitpatcharalert et al. found that 3% of patients developed cardiac disease in the conventional group and 4% in the hypofractionated group [20], thus cardiac events were comparable between the groups. The longer follow-up interval in our second patient cohort did not result in a statistically significant difference for grade 2 or higher late cardiac toxicity between the CF-PMRT and HF-PMRT groups. Another trial from Morocco also reported that no patients developed cardiovascular disease during 64 months of follow-up when using 42 Gy in 15 fractions [16].

With respect to pulmonary fibrosis, almost all of the aforementioned studies reported up to 6% radiation-induced lung fibrosis in both radiotherapy schedules with no statistically significant difference between the groups [13–20]. In the present study, there was statistically significant higher recorded lung symptoms in the HF-PMRT than the CF-PMRT group in the first patient cohort. The grading systems factor in a combination of clinical (RTOG) and radiographic changes (EORTC) were used in the second patient cohort. Although the patchy radiographic changes (EORTC grade 2) or increased density changes (EORTC grade 3) were found much higher in the CF-PMRT than HF-PMRT group (16% vs 9%), but the moderate or persistent lung symptoms requiring symptomatic treatment (RTOG grade > 2) were found no more than 1% in both treatment schemes. This is in line with the review article by Agrawal S [23]. that radiologic lung injury is more common than symptomatic pneumonitis. The classic manifestation in the chest radiographs was the patchy consolidation confined to the radiotherapy field. Chest X-ray abnormalities only with no clinical pneumonitis was reported in 35% in 87 breast cancer patients receiving postoperative radiotherapy [24].

Brachial plexopathy and rib fractures in the supraclavicular area have also been related to radiotherapy toxicity, especially within the junction of the radiation fields, but complications arising from them are rare. Many of the aforementioned researches have reported that no brachial plexopathy and rib fractures were observed in either the conventional or hypofractionated regimens. In the present study, we did not find any severe brachial plexopathy (grade 2) in either PMRT schedule. However, grade 1 brachial plexopathy (mild sensory deficit, no pain, and no treatment required) was found significant higher in CF-PMRT group.

In addition, the incidence of grade 2 or higher lymphedema was approximately 25% in the three different HF-PMRT regimens in the Pakistanis study [13]. The trial in Morocco evaluated grade 2 or higher arm edema in only 5.8% of patients [16]. Khan et al. prospectively reviewed 69 patients using 3.3 Gy with 11 fractions via 3D-conformal radiotherapy (CRT) and found that 4.5% of patients developed grade 2 or higher arm edema [17]. In the Egyptian study [14], grade 2 or higher lymphedema was noted in the conventional group and two hypofractionated groups as 15, 17, and 17%, with no statistically significant differences. In the Greek study [15], no grade 2 lymphedema was seen either in the conventional or hypofractionated groups during the study period. Furthermore, the Chinese randomized trial confirmed a statistically insignificant difference for grade 1–3 lymphedema, which were 21 and 20% in the conventional and hypofractionated groups, respectively [18]. Randomized study from India also reported an insignificant difference rate of grade 2 or higher lymphedema between conventional and hypofractionated radiotherapy (10 and 12%, respectively) [19]. Grade 2 or higher lymphedema had a very low incidence rate in the series from Greece [15] which 15% of their patients received sentinel lymph node biopsy and in the study by Khan et al. [17] which avoid level I axillary irradiation. In the second patient cohort of our study, we used the ISL method [11] to assess the severity of lymphedema, which is different from the other studies [1–7]; lymphedema stage 2 in 4 patients in CF-PMRT (1%) and 4 patients in HF-PMRT (1%) indicated no statistically significant difference between the two groups. All of them received regional nodal irradiation and 5 of them had the radiotherapy field covering the supraclavicular and whole axilla.

The weakness of our study was it being retrospective at a single center. In addition, our data were mixed between 2D and IMRT and most of our patients were treated via the 2D technique. Results from the recent prospective study of HF and CF-PMRT from China [18] confirmed our study outcome that there were no significant differences between the two radiotherapy schedules in terms of the late toxicities.

## Conclusions

This is the largest and longest follow-up study of HF-PMRT for breast cancer compared with CF-PMRT with the data being retrospective from a single institute. We demonstrated similar LRRFS, DFS, and OS for both fractionation schedules. Although there is evidence for a significant increase in grade 2 or more late skin/subcutaneous tissue toxicity which correlated with high proportion of additional radiation dose in HF-PMRT group, HF-PMRT schedule offered fewer moderate grade of late lung and brachial plexus toxicity. Nevertheless, severe grades of all late toxicities were not seen in both radiotherapy regimen. We concluded that alternative HF-PMRT is feasible and safe for clinical application, especially in a country with limited resources.

## Supplementary information

**Additional file 1:** **Table S1.** A comparison of treatment outcomes between CF-PMRT and HF-PMRT.

**Additional file 2:** **Table S2.** A comparison of late toxicity between CF-PMRT and HF-PMRT.

## Data Availability

The datasets used and/or analysed during the current study are available from the corresponding author on reasonable request.

## Abbreviations

AJCC: American Joint Committee on Cancer staging
BED: Biological effective dose
CF: Conventional fractionated
CT: Computerized tomography
DFS: Disease-free survival
EORTC: European Organization for Research and Treatment of Cancer
HF: Hypofractionated
HFRT: Hypofractionated radiotherapy
HT: Helical tomotherapy
IMRT: Intensity-modulated radiotherapy
IQR: Interquartile range
ISL: International Society of Lymphology
LRC: Locoregional control
LRR: Locoregional recurrent rate
LRRFS: Locoregional recurrence-free survival
OS: Overall survival
PMRT: Postmastectomy radiotherapy
RTOG: The Radiation Therapy Oncology Group

## References

[1] Ragaz J, Olivotto IA, Spinelli JJ, Phillips N, Jackson SM, Wilson KS (2005). Locoregional radiation therapy in patients with high-risk breast cancer receiving adjuvant chemotherapy: 20-year results of the British Columbia randomized trial. J Natl Cancer Inst.

[2] Nielsen HM, Overgaard M, Grau C, Jensen AR, Overgaard J, Danish Breast Cancer Cooperative G (2006). Study of failure pattern among high-risk breast cancer patients with or without postmastectomy radiotherapy in addition to adjuvant systemic therapy: long-term results from the Danish Breast Cancer Cooperative Group DBCG 82 b and c randomized studies. J Clin Oncol.

[3] Clarke MCR, Darby S, Davies C, Davies C, Elphinstone P, Evans V (2005). Effects of radiotherapy and of differences in the extent of surgery for early breast cancer on local recurrence and 15-year survival: an overview of the randomised trials. Lancet..

[4] McGale P, Taylor C, Correa C, Cutter D, Duane F, EBCTCG (Early Breast Cancer Trialists’ Collaborative Group) (2014). Effect of radiotherapy after mastectomy and axillary surgery on 10-year recurrence and 20-year breast cancer mortality: meta-analysis of individual patient data for 8135 women in 22 randomised trials. Lancet.

[5] Thames HDBS, Turesson I, Overgaard M, Van den Bogaert W (1990). Time-dose factors in radiotherapy: a review of the human data. Radiother Oncol.

[6] Whelan TJ, Pignol JP, Levine MN, Julian JA, MacKenzie R, Parpia S (2010). Long-term results of hypofractionated radiation therapy for breast cancer. N Engl J Med.

[7] Bentzen SM, Agrawal RK, Aird EG, Barrett JM, Barrett-Lee PJ, START Trialists group (2008). The UK Standardisation of Breast Radiotherapy (START) Trial A of radiotherapy hypofractionation for treatment of early breast cancer: a randomised trial. Lancet Oncol.

[8] Bentzen SM, Agrawal RK, Aird EG, Barrett JM, Barrett-Lee PJ, START Trialists group (2008). The UK Standardisation of Breast Radiotherapy (START) Trial B of radiotherapy hypofractionation for treatment of early breast cancer: a randomised trial. Lancet.

[9] Haviland JS, Owen JR, Dewar JA, Agrawal RK, Barrett J, Barrett-Lee PJ (2013). The UK Standardisation of Breast Radiotherapy (START) trials of radiotherapy hypofractionation for treatment of early breast cancer: 10-year follow-up results of two randomised controlled trials. Lancet Oncol.

[10] Cox JD, Stetz J, Pajak TF (1995). Toxicity criteria of the Radiation Therapy Oncology Group (RTOG) and the European Organization for Research and Treatment of Cancer (EORTC). Int J Radiat Oncol Biol Phys.

[11] International Society of Lymphology (2013). The diagnosis and treatment of peripheral lymphedema: 2013 consensus document of the International Society of Lymphology. Lymphology..

[12] Bajrovic A, Rades D, Fehlauer F, Tribius S, Hoeller U, Rudat V (2004). Is there a life-long risk of brachial plexopathy after radiotherapy of supraclavicular lymph nodes in breast cancer patients?. Radiother Oncol.

[13] Shahid A, Athar MA, Asghar S, Zubairi T, Murad S, Yunas N (2009). Post mastectomy adjuvant radiotherapy in breast cancer: a comparision of three hypofractionated protocols. J Pak Med Assoc.

[14] Eldeeb H, Awad I, Elhanafy O (2012). Hypofractionation in post-mastectomy breast cancer patients: seven-year follow-up. Med Oncol.

[15] Kouloulias V, Mosa E, Zygogianni A, Kypraiou E, Georgakopoulos J, Platoni K (2016). A retrospective analysis of toxicity and efficacy for 2 hypofractionated irradiation schedules versus a conventional one for post-mastectomy adjuvant radiotherapy in breast cancer. Breast Care.

[16] Bellefqih S, Elmajjaoui S, Aarab J, Khalil J, Afif M, Lachgar A (2017). Hypofractionated regional nodal irradiation for women with node-positive breast cancer. Int J Radiat Oncol Biol Phys.

[17] Khan AJ, Poppe MM, Goyal S, Kokeny KE, Kearney T, Kirstein L (2017). Hypofractionated postmastectomy radiation therapy is safe and effective: first results from a prospective phase II trial. J Clin Oncol.

[18] Wang SL, Fang H, Song YW, Wang WH, Hu C, Liu YP (2019). Hypofractionated versus conventional fractionated postmastectomy radiotherapy for patients with high-risk breast cancer: a randomised, non-inferiority, open-label, phase 3 trial. Lancet Oncol.

[19] Rastogi K, Jain S, Bhatnagar AR, Bhaskar S, Gupta S, Sharma N (2018). A comparative study of hypofractionated and conventional radiotherapy in postmastectomy breast cancer patients. Asia Pac J Oncol Nurs.

[20] Pinitpatcharalert A, Chitapanarux I, Euathrongchit J, Tharavichitkul E, Sukthomya V, Lorvidhaya V (2011). A retrospective study comparing hypofractionated radiotherapy and conventional radiotherapy in postmastectomy breast cancer. J Med Assoc Thail.

[21] Ordonez-Sanz C, Bowles S, Hirst A, MacDougall ND (2014). A single plan solution to chest wall radiotherapy with bolus?. Br J Radiol.

[22] Vu TT, Pignol JP, Rakovitch E, Spayne J, Paszat L (2007). Variability in radiation oncologists’ opinion on the indication of a bolus in post-mastectomy radiotherapy: an international survey. Clin Oncol (R Coll Radiol).

[23] Agrawal S (2013). Clinical relevance of radiation pneumonitis in breast cancers. South Asian J Cancer.

[24] Rancati T, Wennberg B, Lind P, Svane G, Gagliardi G (2007). Early clinical and radiological pulmonary complications following breast cancer radiation therapy: NTCP fit with four different models. Radiother Oncol.

