# Supplementary material for: Conventional versus hypofractionated postmastectomy radiotherapy: a report on long-term outcomes and late toxicity
Source: Radiat Oncol. 2019 Oct 14;14:175. doi: 10.1186/s13014-019-1378-x (PMC6790998; doi:10.1186/s13014-019-1378-x)
Supplement: Supplementary file 1 — Additional file 1: Table S1. A comparison of treatment outcomes between CF-PMRT and HF-PMRT. [file 13014_2019_1378_MOESM1_ESM.docx]

Additional file 1

Table S1. A comparison of treatment outcomes between CF-PMRT and HF-PMRT

| Study | Duration | Design | n | Median FU  (months) | Technique | Schedule | OS | DFS | LRR | LRC | DM | DMFS |
| --- | --- | --- | --- | --- | --- | --- | --- | --- | --- | --- | --- | --- |
| Shahid et al.[13] | 1998–2004 | Randomized | 300 | 60 | 2D | 27Gy/5F | NA | 83% | 11% | NA | 26% | NA |
|  |  |  |  |  |  | 35Gy/10F |  | 82% | 12% |  | 24% |  |
|  |  |  |  |  |  | 40Gy/15F |  | 80% | 10% |  | 28% |  |
| Eldeeb et al[14] | 2001–2004 | Retrospective | 107 | 23 | 2D | 50Gy/25F | 90% | NA | 7% | NA | NA | NA |
|  |  |  |  |  |  | 45Gy/17F | 94% |  | 3% |  |  |  |
|  |  |  |  |  |  | 40Gy/15F | 90% |  | 7% |  |  |  |
| Kouloulias et al.[15] | 2008–2011 | Retrospective | 117 | 36 | 3D-CRT | 50Gy/25F | NA | NA | 0% | NA | NA | NA |
|  |  |  |  |  |  | 48.3Gy/21F |  |  | 0% |  |  |  |
|  |  |  |  |  |  | 42.6Gy/16F |  |  | 0% |  |  |  |
| Bellefquih et al [16] | 2009–2011 | Retrospective | 257 | 64 | 2D | 42Gy/15F | 87% | 84% | NA | 94% | NA | 83% |
| Khan et al. [17] | 2010–2014 | Prospective | 69 | 32 | 3D-CRT | 36.6Gy/11F | NA | NA | 3% | 89% | NA | 90% |
| Wang et al. [18] | 2008–2016 | Randomized | 820 | 58.5 | 2D | 50Gy/25F | 86% | 70% | 8.1% | NA | 27% | NA |
|  |  |  |  |  |  | 43.5Gy/15F | 84% | 74% | 8.3% |  | 23% |  |
| Rastogi K, [19] | 2014-2017 | Randomized | 100 | 20 | 3D-CRT | 50Gy/25F | 100% | 94% | 2% | NA | 4% | NA |
|  |  |  |  |  |  | 42.7Gy/16F | 100% | 90% | 2% | NA | 8% | NA |
| Pinitpatcharalert et al. [20] | 2004–2006 | Retrospective | 215 | 39 | 2D | 50Gy/25F | 63% | 63% | NA | 87% | NA | NA |
|  |  |  |  |  |  | 42–47.7Gy/16–18F | 73% | 70% |  | 86% |  |  |
| Our study  (2^nd^ cohort) | 2004–2014 | Retrospective | 998 | 71.8 | 2D or IMRT | 50–60Gy/25–30F | 74% | 72% | NA | 94% | NA | NA |
|  |  |  |  |  |  | 42.4–53Gy/16–20F | 73% | 70% |  | 96% |  |  |

CF-PMRT = Conventional fractionated post mastectomy radiotherapy; HF-PMRT = Hypofractionated post mastectomy radiotherapy; N = number ; NA = not applicable; OS = overall survival rate; DFS = disease free survival rate; LRR = locoregional recurrent rate; LRC = locoregional control rate; DM = distant metastasis rate; DMFS = distant metastasis free survival rate; 2D = 2 dimension, 3D-CRT = 3 dimension conformal radiotherapy; IMRT = intensity-modulated radiotherapy; Gy = Gray; F = fractions.
