# Supplementary material for: Conventional versus hypofractionated postmastectomy radiotherapy: a report on long-term outcomes and late toxicity
Source: Radiat Oncol. 2019 Oct 14;14:175. doi: 10.1186/s13014-019-1378-x (PMC6790998; doi:10.1186/s13014-019-1378-x)
Supplement: Supplementary file 2 — Additional file 2: Table S2. A comparison of late toxicity between CF-PMRT and HF-PMRT. [file 13014_2019_1378_MOESM2_ESM.docx]

Additional file 2

Table S2. A comparison of late toxicity between CF-PMRT and HF-PMRT

| Study | Schedule | Toxicity | | | | | |
| --- | --- | --- | --- | --- | --- | --- | --- |
|  |  | Skin | Heart | Lung | Brachial Plexopathy | Lymphedema | Rib Fracture |
| Shahid et al.[13] | 27 Gy/5F | NA | 5% LVEF drop | 4% | 0% | gr>2;21% | 0% |
|  | 35 Gy/10F |  | 6% LVEF drop | 5% | 0% | gr>2;22% | 0% |
|  | 40 Gy/15F |  | 5% LVEF drop | 5% | 0% | gr>2;27% | 0% |
| Eldeeb et al[14] | 50 Gy/25F | gr>2;17% | NA | NA | NA | gr>2;15% | NA |
|  | 45 Gy/17F | gr>2;33% |  |  |  | gr>2;17% |  |
|  | 40 Gy/15F | gr>2;37% |  |  |  | gr>2;17% |  |
| Kouloulias et al.[15] | 50 Gy/25F | gr>2;0% | NA | gr>2 ;3.3% | 0% | gr>2;0% | 0% |
|  | 48 Gy/21F | gr>2;0% |  | gr>2 ;1.7% | 0% | gr>2;0% | 0% |
|  | 43 Gy/16F | gr>2;0% |  | gr>2 ;3.7% | 0% | gr>2;0% | 0% |
| Bellefquih et al [16] | 42 Gy/15F | 30.7% | 0% | 0% | 0% | gr>2;5.8% | 0% |
| Khan et al. [17] | 37 Gy/11F | gr2;1.4% | NA | NA | NA | gr2;4.5% | NA |
| Wang et al. [18] | 50 Gy/25F | gr3; 0% | 1% | 0% | 0% | gr1-3;20.5% | 0% |
|  | 44 Gy/15F | gr3; 0.2% | 2% | 0% | 0% | gr1-3;20.2% | 0% |
| Rastogi K, [19] | 50Gy/25F | gr>2;4% | NA | gr>2;6% | NA | gr>2;10% | NA |
|  | 43Gy/16F | gr>2;4% | NA | gr>2;2% | NA | gr>2;12% | NA |
| Pinitpatcharalert et al. [20] | 50 Gy/25F | gr>2;9.1% | 3% | gr>2; 0% | gr>2;3.1% | gr>2;3.3% | 0% |
|  | 42–48 Gy/16–18F | gr>2;10.2% | 4% | gr>2; 0% | gr>2;7.8% | gr>2;3.8% | 1% |
| Our study  (2^nd^ cohort) | 50–60 Gy/25–30F | Skin gr >2; 1%  Subcutaneous tissue >2; 2% | gr >2; <1% | Clinical  gr >2; 1%  Imaging gr≥2; 16% | Gr 2; 0% | Gr 2; 1% | NA |
|  | 42–53 Gy/16–20F | Skin gr >2; 4%  Subcutaneous tissue >2; 7% | gr >2; <1% | Clinical  gr >2; <1%  Imaging gr≥2; 9% | Gr 2; 0% | Gr 2; 1% | NA |

CF-PMRT = Conventional fractionated post mastectomy radiotherapy; HF-PMRT = Hypofractionated post mastectomy radiotherapy; NA = not applicable; LVEF = left ventricular ejection fraction; gr = grade.
